# Inhibition of Adipocyte Lipolysis Reduces Liver Injury in a Mouse Model of Ischemia Reperfusion Injury

**DOI:** 10.1016/j.jcmgh.2025.101679

**Published:** 2025-11-19

**Authors:** Kim H.H. Liss, Samantha Goldman, Mai He, Trevor M. Shew, Daniel Ferguson, Brian N. Finck

**Affiliations:** 1 Department of Pediatrics, Washington University School of Medicine, St. Louis, Missouri; 2 Department of Medicine, Washington University School of Medicine, St. Louis, Missouri; 3 Department of Pathology and Immunology, Washington University School of Medicine, St. Louis, Missouri

**Keywords:** ATGL, Fatty Acids, Lipids, Liver transplantation

## Abstract

**Background & Aims:**

Hepatic ischemia reperfusion injury (IRI) is unavoidable in most liver operations and is associated with poor patient and graft outcomes in the setting of liver transplantation. However, there are no pharmacological interventions available for treatment of IRI. Prior work has demonstrated that liver IRI leads to hepatic lipid accumulation, suggesting that increased adipocyte lipolytic rates may contribute to hepatic steatosis. Inhibition of adipose triglyceride lipase (ATGL), the rate-limiting enzyme involved in triglyceride hydrolysis, may be beneficial in cardiac injury and alcoholic liver disease, but its role in liver IRI has not been investigated. Our objective was to assess the effects of inhibition of adipose tissue lipolysis in the setting of liver IRI.

**Methods:**

Wild-type mice were treated with Atglistatin, a small molecule inhibitor of ATGL, prior to IRI. Mice with hepatocyte- or adipocyte-specific deletion of *Pnpla2*, the gene encoding ATGL, were generated and subjected to a mouse model of IRI. Mouse hepatocytes were cultured with fatty acids in an in vitro model of IRI.

**Results:**

We demonstrated that experimental IRI was associated with increased adipocyte lipolysis. Pharmacological and genetic inhibition of adipocyte lipolysis reduced plasma and hepatic free fatty acids and decreased circulating transaminases and liver inflammation following hepatic IRI. Furthermore, exogenous fatty acids were sufficient to increase cell death and the expression of inflammatory cytokines in in vitro IRI.

**Conclusions:**

These data suggest that targeting adipocyte lipolysis may represent a novel therapeutic approach in the prevention of hepatic IRI, which could improve patient and graft outcomes following liver transplantation.

**Summary**

Hepatic ischemia reperfusion injury (IRI) is associated with poor organ function in the setting of transplantation. Genetic and pharmacological inhibition of adipocyte lipolysis attenuates IRI. Thus, targeting adipocyte lipolysis may represent a novel therapeutic approach to reducing hepatic IRI.

**What You Need to Know**

**Background**

There are no pharmacological interventions to attenuate hepatic ischemia reperfusion injury (IRI). Our objective was to assess the effects of inhibiting adipose tissue lipolysis in the setting of liver IRI.

**Impact**

Inhibition of adipocyte lipolysis attenuates IRI. These animal studies identify a novel role for adipose-liver cross talk and suggest that inhibiting lipolysis represents a new therapeutic target to reduce IRI.

**Future Directions**

Future studies should elucidate the role of lipolysis in people undergoing liver transplantation. Inhibition of lipolysis by pharmacological or nutritional interventions has the potential to improve patient and graft outcomes.

This article has an accompanying editorial.

Hepatic ischemia reperfusion injury (IRI) results from temporary interruption of blood supply followed by reestablishment of blood flow to the liver. Some degree of IRI is unavoidable in most liver operations, which leads to organ injury. In the setting of liver transplantation, severe IRI can result in primary nonfunction or early allograft dysfunction. IRI is characterized by an initial ischemic phase that causes cell death, followed by reperfusion, during which an overly robust inflammatory response causes the majority of organ injury.^1^ Clinically, IRI is associated with inferior patient and graft outcomes in liver transplantation.^2^, ^3^, ^4^ Importantly, there are currently no pharmacological interventions available to prevent or treat IRI.

Few studies have evaluated the role of interorgan cross talk in hepatic IRI. Adipose tissue plays an important role in systemic energy homeostasis, and adipose tissue dysfunction has been associated with multiple disease states including metabolic dysfunction-associated liver disease (MASLD).^5^^,^^6^ In a fed state, excess energy is stored as triglycerides (TAG) within adipocyte lipid stores. Lipolytic stimuli, such as starvation, cold exposure, and norepinephrine, enhance lipolysis, which occurs via sequential hydrolysis of TAG by adipose triglyceride lipase (ATGL), hormone-sensitive lipase (HSL), and monoglyceride lipase (MGL) resulting in the release of free fatty acids (FFAs) and glycerol into the blood. The rate limiting step in adipose tissue lipolysis is catalyzed by ATGL (encoded by patatin-like phospholipase domain containing 2 [*Pnpla2*]), which hydrolyzes TAG to generate diacylglycerol (DAG).^7^ Lipolysis is regulated post-translationally by protein kinase A-mediated phosphorylation of multiple lipolytic mediators. ATGL activity is also regulated by co-factors including comparative gene identification 58 (CGI-58; encoded by *Abhd5*), G0/G1 switch gene 2 (G0S2), and hypoxia-inducible lipid droplet associated protein (HILPDA), encoded by hypoxia inducible gene 2 [*Hig2*]).^7^ People with mutations in *PNPLA2* develop neutral lipid storage disease, characterized by accumulation of TAG in muscle and other tissues leading to progressive myopathy, cardiomyopathy, and diabetes.^8^, ^9^, ^10^ Mouse models with global or cell-specific deletion of *Pnpla2*, and small molecule ATGL inhibitors, have highlighted the importance of ATGL in regulating systemic metabolism and its involvement in metabolic disease.^11^, ^12^, ^13^, ^14^ FFAs released into the blood by adipose tissue are avidly taken up by the liver, and acute activation of adipocyte lipolysis can significantly alter the hepatic lipidome and transcriptome.^15^^,^^16^ Adipose lipolysis also plays a critical role in the development of steatosis in alcoholic liver disease^17^ and MASLD.^6^ In addition, adipocyte lipolysis has been shown to contribute to cardiac injury in mouse IRI models, and inhibition of lipolysis attenuates injury in this setting.^18^

We have previously found IRI to be associated with significant and dynamic changes in the hepatic lipidome.^19^ However, whether adipocyte lipolysis contributes to alterations in hepatic lipid dynamics and IRI injury is unclear. In this study, we found that adipose tissue lipolysis is increased in a mouse model of warm hepatic IRI. We then tested the hypothesis that inhibition of adipocyte lipolysis would attenuate hepatic IRI. Using pharmacological and genetic approaches to inhibit ATGL, we found that inhibition of adipocyte lipolysis mitigates liver injury after IRI, whereas hepatocyte-specific deletion of ATGL did not. Collectively, these studies identify a novel role for adipose-liver cross talk and suggest that inhibiting lipolysis by pharmacological or nutritional interventions represent a promising new therapeutic target to reduce liver IRI.

## Results

### Hepatic Ischemia Reperfusion Injury Is Associated With Adipose Tissue Lipolysis

**Figure 1:**
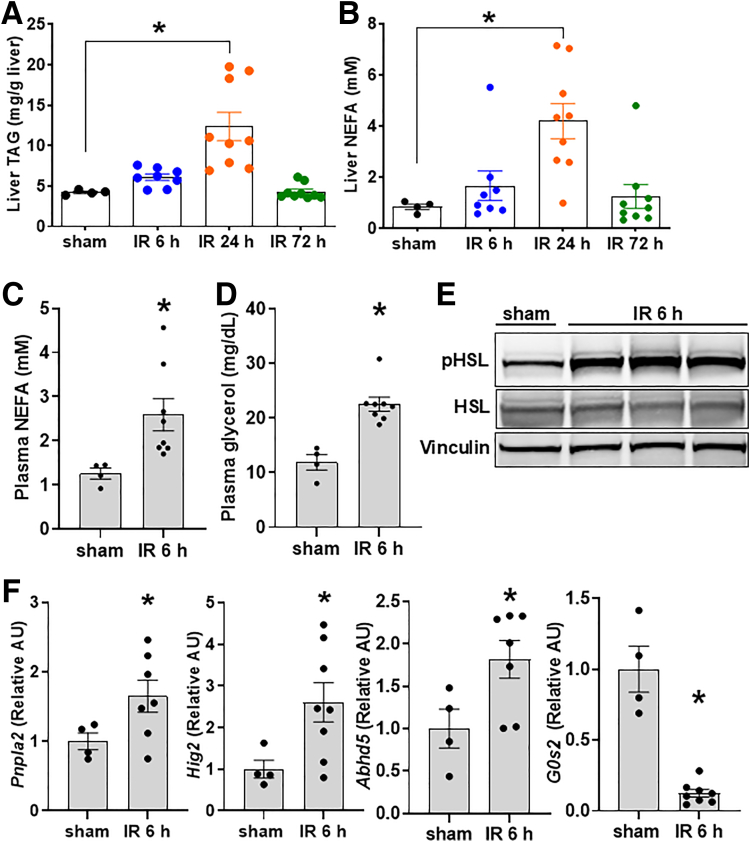
**Hepatic IRI is associated with adipose tissue lipolysis.** (*A*) Liver TAG content following sham operation or IR surgery. (*B*) Liver NEFA concentration following sham or IR surgery. (*C*) Plasma NEFA concentration following sham or IR surgery. (*D*) Plasma glycerol concentration following sham or IR surgery. (*E*) Adipose tissue (eWAT) protein expression of phosphorylated HSL, HSL, and vinculin after sham or IR surgery. (*F*) Adipose tissue (eWAT) gene expression of *Pnpla2*, *Hig2*, Abhd5, and *G0s2*. Values are mean ± SEM. n = 4 per sham group, 8–10 per IR surgery group. Sham indicates sham surgery. 6 h, 24 h, 72 h indicates hours of reperfusion following IR surgery. ∗*P* < .05.

Our prior work demonstrated that, even in lean mice on a standard chow diet, hepatic IRI led to the accumulation of lipids in liver 24 hours later.^19^ We have found that liver triacylglycerol (TAG) and non-esterified free fatty acids (NEFAs) accumulation likely begins at earlier time points but becomes significantly increased compared with sham-operated animals at 24 hours following reperfusion. This is followed by normalization by 72 hours reperfusion (Figure 1*A, B*). To investigate whether lipolysis contributed to this phenomenon after hepatic IRI, we first performed IR surgery in chow-fed C57BL/6J mice and examined plasma and adipose tissue markers of lipolysis at the 6-hour timepoint to identify changes proceeding the development of steatosis. We found that plasma NEFA and free glycerol, which are released by adipose tissue through lipolysis, were increased 6 hours after hepatic IRI relative to sham operated animals at the same timepoint (Figure 1*C, D*). In addition, we found elevated adipose tissue phosphorylation of HSL following IR surgery compared with sham operated animals (Figure 1*E*). Adipose tissue expression of genes involved in enhanced lipolysis (*Pnpla2, Hig2,* and *Abhd5*) were increased, whereas expression of the lipolytic inhibitor *G0s2*, was decreased (Figure 1*F*). Taken together, these data suggest that hepatic IRI is associated with increased adipocyte lipolysis, and these metabolic changes precede the development of hepatic steatosis observed at 24 hours following IRI.^19^

### Pharmacological Inhibition of ATGL Attenuates Hepatic IRI

**Figure 2:**
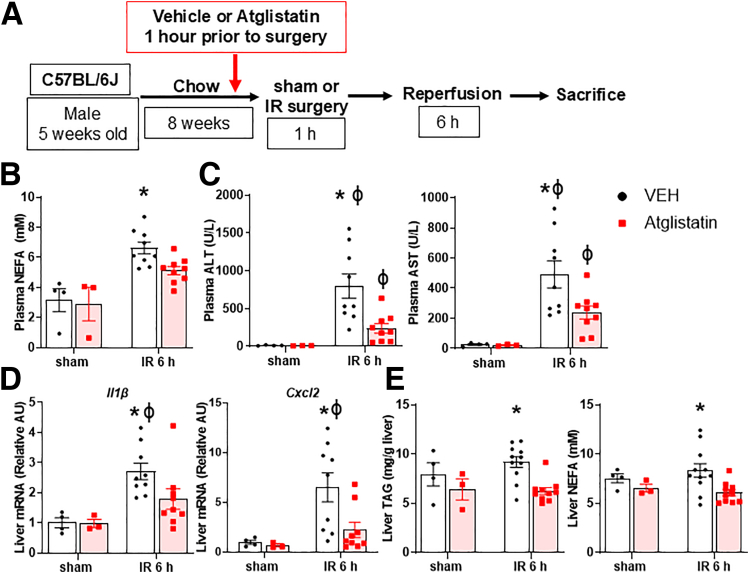
**Pharmacological inhibition of ATGL attenuates hepatic IRI.** (*A*) Schematic detailing experimental design. Mice were either treated with vehicle (VEH; olive oil) or Atglistatin (100 um/kg) by oral gavage an hour prior to sham or IR surgery. Mice were then recovered for 6 hours before sacrifice, at which time blood and tissue were collected for analysis. (*B*) Plasma NEFA following sham or IR surgery in vehicle- or Atglistatin-treated mice. (*C*) Plasma ALT and AST following sham or IR surgery in vehicle- or Atglistatin-treated mice. (*D*) Liver gene expression of *Il1β* and *Cxcl2*. (*E*) Liver TAG and NEFA concentration following sham or IR surgery in vehicle- or Atglistatin-treated mice. Values are mean ± SEM. n = 3–4 per sham group, 8 per IR surgery group. Sham indicates sham surgery. 6 h indicates hours of reperfusion following IR surgery. ∗*P* < .05 between VEH and Atglistatin mice at the same time point. ^ϕ^*P* < .05 between VEH sham vs VEH 6 hours or Atglistatin sham vs Atglistatin 6 hours.

To determine if inhibition of lipolysis would attenuate hepatic IRI, we treated mice with either a small molecule inhibitor of ATGL, Atglistatin, or olive oil vehicle, 1 hour before sham or IR surgery (Figure 2*A*). Atglistatin is a highly selective, direct inhibitor of ATGL with no activity against MGL or HSL.^20^ Furthermore, Atglistatin does not alter the expression or phosphorylation of ATGL or its co-activators/repressors, is quick acting, has a short half-life, and does not lead to ectopic lipid accumulation even in the setting of chronic administration.^12^ Following hepatic IRI, plasma NEFA increased in vehicle-treated mice, but this response was significantly blunted in Atglistatin-treated mice, suggesting that Atglistatin treatment reduces lipolysis in the setting of liver IRI (Figure 2*B*). Compared with vehicle treatment, mice treated with Atglistatin exhibited significantly decreased plasma alanine transaminase (ALT) and aspartate transaminase (AST), which are biomarkers of liver injury, at 6 hours after IR surgery (Figure 2*C*). We then measured liver gene expression of cytokines and chemokines and found a significant reduction in the expression of *Il1β* and *Cxcl2* in Atglistatin-treated mice compared with vehicle-treated mice (Figure 2*D*). Interestingly, after 1 dose of Atglistatin, liver TAG and NEFA content also significantly decreased compared with vehicle-treated mice following IRI (Figure 2*E*). Collectively, these findings suggest that pharmacological inhibition of ATGL reduced plasma NEFA and hepatic steatosis and attenuated liver injury in the setting of IR surgery.

### Hepatocyte-specific Deletion of ATGL Does Not Attenuate Hepatic IRI

**Figure 3:**
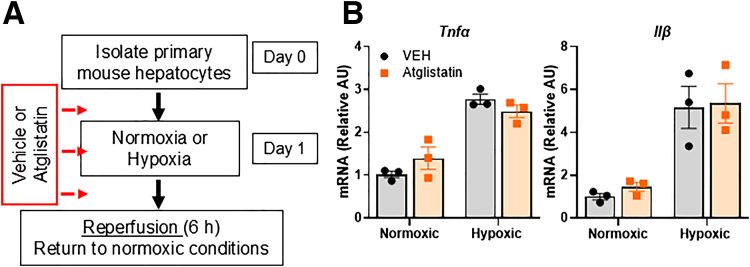
**Atglistatin does not decrease inflammation in primary hepatocytes exposed to in vitro IRI.** (*A*) Schematic detailing in vitro ischemia reperfusion injury. Primary hepatocytes were isolated from C57BL/6J mice. After overnight culture, cells were treated with vehicle (DMSO; VEH) or Atglistatin immediately before exposure to either normoxic or hypoxic conditions and during reperfusion. (*B*) Hepatocyte gene expression of *Tnfα* and *Il1β* at time of reperfusion in cells treated with vehicle or Atglistatin. Values are mean ± SEM. n = 3 separate experiments, 3 wells per condition.

**Figure 4:**
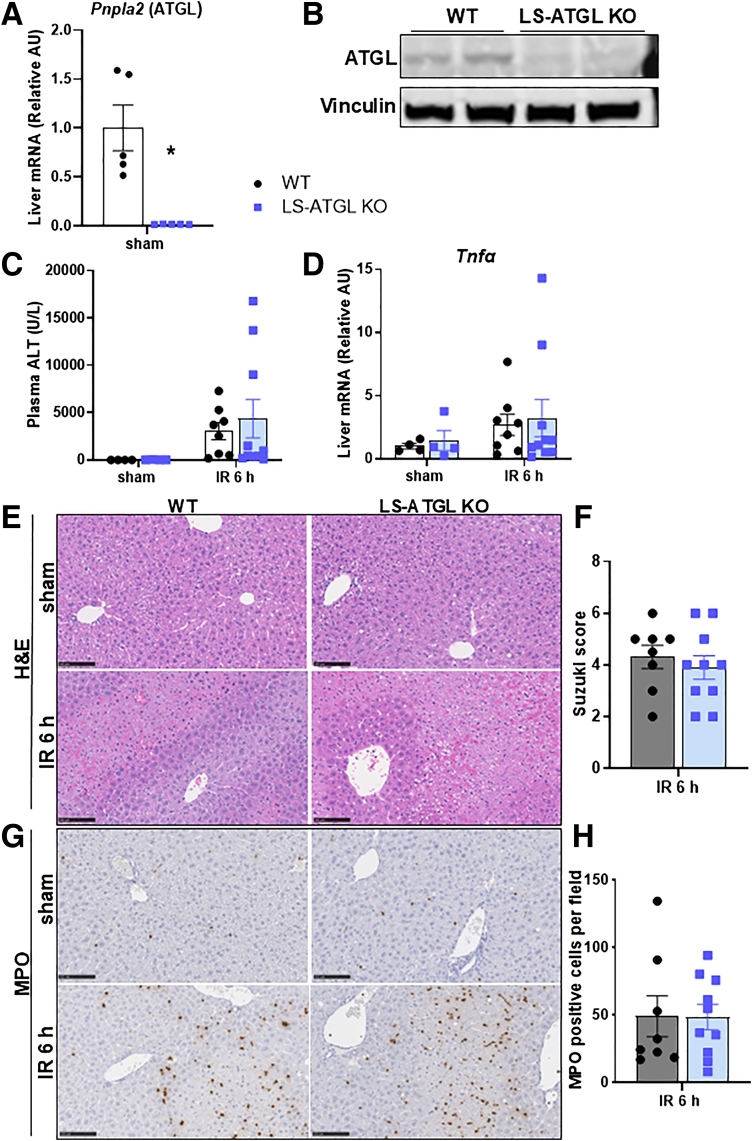
**Hepatocyte-specific deletion of ATGL does not protect against hepatic IRI.** (*A*) Liver gene expression of *Pnpla2* (gene encoding ATGL) in hepatocyte-specific ATGL KO mice (LS-ATGL KO) and WT littermates (WT). (*B*) Liver protein expression of ATGL and vinculin in WT and LS-ATGL KO mice. (*C*) Plasma ALT in WT and LS-ATGL KO mice following sham or IR surgery with 6 hours of reperfusion. (*D*) Liver gene expression of *Tnfα* in WT and LS-ATGL KO mice following sham or IR surgery with 6 hours of reperfusion. (*E*) Representative images of H&E-stained liver sections in WT and LS-ATGL KO mice following sham or IR surgery with 6 hours of reperfusion. (*F*) Suzuki scores based on H&E sections in WT and LS-ATGL KO mice after IR surgery and 6 hours of reperfusion. (*G*) Representative images from liver sections stained with MPO in WT and LS-ATGL KO mice following sham or IR surgery and 6 hours of reperfusion. (*H*) Quantitative scoring of MPO staining in WT and LS-ATGL KO mice after IR surgery and 6 hours of reperfusion. Values are mean ± SEM. n = 4 per sham group, 8–10 per IR surgery group. Sham indicates sham surgery. 6 h indicates hours of reperfusion following IR surgery. ∗*P* < .05 between WT and KO mice at the same time point.

Atglistatin can accumulate in adipose and liver tissue and lead to a decrease of ATGL activity in both tissue types.^20^ Thus, it was unclear whether Atglistatin has a direct effect on the liver or if it reduces hepatic IRI by inhibiting adipose tissue ATGL. We therefore isolated primary mouse hepatocytes from C57BL/6J mice and exposed them to IRI in vitro. Before, during, and after ischemia, we treated hepatocytes with vehicle or Atglistatin (Figure 3*A*). After 5 hours of ischemia and 5 hours of reperfusion, there was no significant difference in hepatocyte expression of inflammatory cytokines (*Tnfα* and *Il1β*) between vehicle- and Atglistatin-treated cells (Figure 3*B*). Next, we generated mice with hepatocyte-specific deletion of ATGL by crossing mice expressing Cre under the control of the albumin promoter with *Pnpla2* floxed mice (LS-ATGL KO). We confirmed there was decreased ATGL gene and protein expression in LS-ATGL KO mice compared with wild-type (WT) littermates (Figure 4*A, B*). Following IR surgery and 6 hours of reperfusion, we observed no difference in histological appearance of necrosis, neutrophil infiltration, plasma markers of liver injury, or hepatic gene expression of inflammatory cytokines between WT and LS-ATGL KO mice (Figure 4*C–H*). Together, these data suggest that the beneficial effects of Atglistatin in IRI are not mediated by inhibition of hepatocyte ATGL.

### Adipocyte-specific Deletion of ATGL Attenuates Hepatic IRI

**Figure 5:**
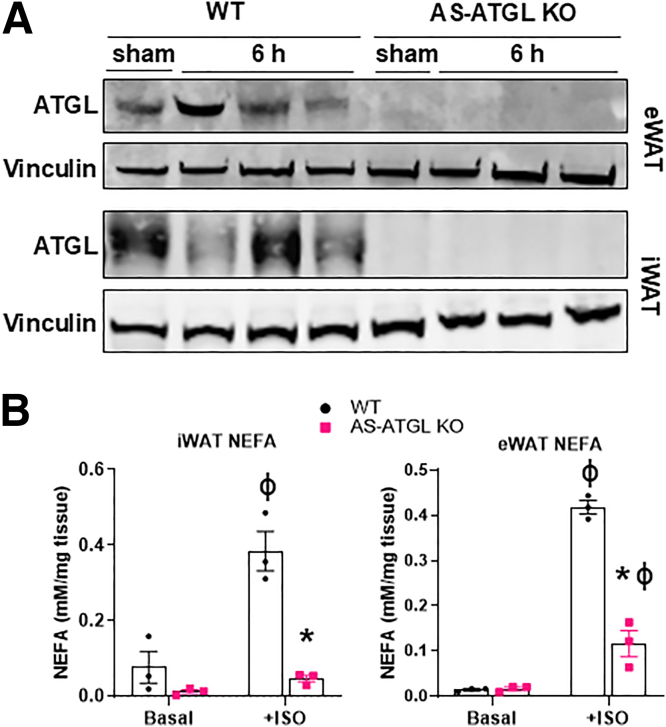
**Adipocyte-specific deletion of ATGL reduces adipocyte lipolysis.** (*A*) Protein expression of ATGL in eWAT and iWAT in adipocyte-specific ATGL knockout (AS-ATGL KO) mice and WT littermates. (*B*) Media NEFA concentration in iWAT and eWAT tissue explants from AS-ATGL KO and WT mice exposed to vehicle (basal) or isoproterenol (ISO) to stimulate lipolysis ex vivo. Values are mean ± SEM. n = 3 separate experiments, 3 wells per condition. ∗*P* < .05 between WT and KO mice under the same condition. ^ϕ^*P* < .05 between WT basal vs WT ISO or KO basal vs KO ISO.

**Figure 6:**
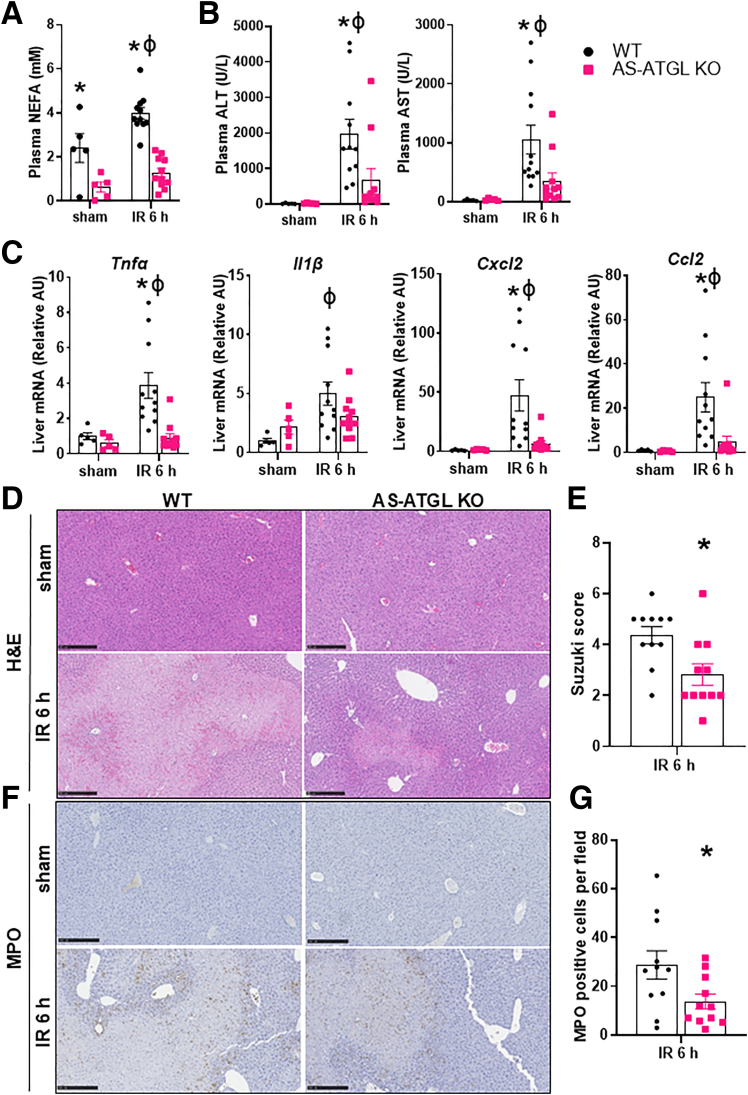
**Adipocyte-specific deletion of ATGL attenuates hepatic IRI.** (*A*) Plasma NEFA in WT and AS-ATGL KO mice following sham or IR surgery and 6-hour reperfusion. (*B*) Plasma ALT and AST in WT and AS-ATGL KO mice following sham or IR surgery and 6-hour reperfusion. (*C*) Liver gene expression of *Tnfα, Il1β, Cxcl2, Ccl2* in WT and AS-ATGL KO mice following sham or IR surgery and 6-hour reperfusion. (*D*) Representative images from liver sections stained with H&E in WT and AS-ATGL KO mice following sham or IR surgery and 6-hour reperfusion. (*E*) Suzuki scores based on H&E sections in WT and AS-ATGL KO mice after IR surgery and 6-hour reperfusion. (*F*) Representative images from liver sections stained with MPO in WT and AS-ATGL KO mice following sham or IR surgery and 6-hour reperfusion. (*G*) Quantitative scoring of MPO staining in WT and AS-ATGL KO mice after IR surgery and 6-hour reperfusion. Values are mean ± SEM. n = 5 per sham group, 11 per IR surgery group. Sham indicates sham surgery. 6 h indicates hours of reperfusion following IR surgery. ∗*P* < .05 between WT and KO mice at the same time point. ^ϕ^*P* < .05 between WT sham vs WT 6 h or KO sham vs KO 6 h.

To determine if specific inhibition of adipocyte lipolysis would attenuate hepatic IRI, we generated mice with adipocyte-specific deletion of ATGL by crossing *Pnpla2* floxed mice (AS-ATGL KO) with mice expressing Cre under the control of the adiponectin promoter. We confirmed that ATGL was undetectable in epididymal (eWAT) and inguinal (iWAT) white adipose tissue (Figure 5*A*). We also verified that ATGL deletion blocked lipolysis, as evidenced by reduced media NEFA levels in WAT explants stimulated with isoproterenol in AS-ATGL KO relative to WT littermates (Figure 5*B*). Next, we subjected AS-ATGL KO and WT littermate mice to liver IR surgery. Following hepatic IRI, plasma NEFA increased in WT mice, but this was significantly blunted in AS-ATGL KO mice (Figure 6*A*). Plasma ALT and AST activity were substantially lower in AS-ATGL KO mice compared with WT mice at 6 hours of reperfusion (Figure 6*B*). Liver inflammatory cytokine and chemokine expression were significantly lower in AS-ATGL KO mice compared with WT mice following hepatic IRI (Figure 6C). Histological examination revealed decreased congestion, vacuolization, and necrosis as measured by Suzuki’s score and decreased neutrophil infiltration (myeloperoxidase [MPO] stain) in AS-ATGL KO mice compared with WT mice (Figure 6*D–G*). Taken together, these data demonstrate that adipocyte-specific deletion of ATGL attenuates liver IRI.

### Adipocyte-specific Deletion of ATGL Significantly Alters the Hepatic Lipidome

**Figure 7:**
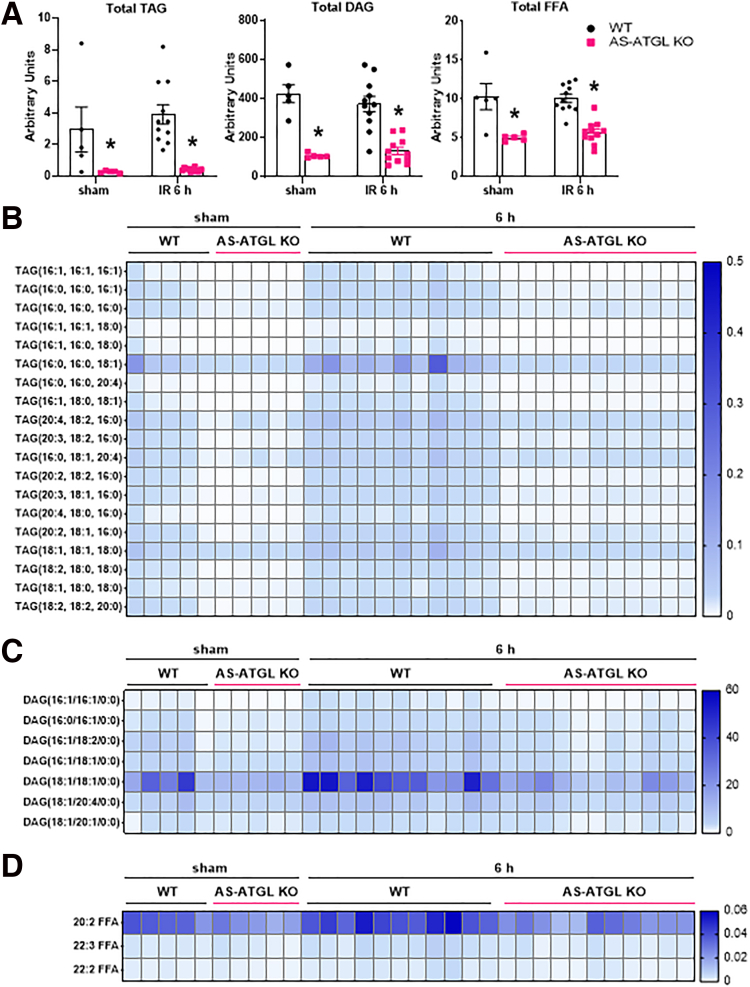
**AS-ATGL KO mice have altered hepatic lipid content following sham and IRI.** (*A*) Liver TAG, DAG, and FFA content in WT and AS-ATGL KO mice following sham or IR surgery and 6-hour reperfusion. (*B*) Liver TAG species noted to be increased (*P* < .05) following IRI in WT mice compared with WT sham. (*C*) Liver DAG species noted to be increased (*P* < .05) following IRI in WT mice compared with WT sham. (*D*) Liver FFA species noted to be increased (*P* < .05) following IRI in WT mice compared with WT sham. Values are mean ± SEM. n = 5 per sham group, 11 per IR surgery group. Sham indicates sham surgery. 6 h indicates hours of reperfusion following IR surgery. ∗*P* < .05 between WT and KO mice at the same time point. ^ϕ^*P* < .05 between WT sham vs WT 6 h or KO sham vs KO 6 h.

Because we have previously observed that hepatic IRI is associated with dynamic changes in the hepatic lipidome across multiple lipid classes,^19^ we measured several lipid species from AS-ATGL KO and WT mice subjected to liver IRI. We found a substantial decrease in hepatic total TAG, DAG, and FFA content in AS-ATGL KO mice compared with WT mice undergoing either sham or IR surgery (Figure 7*A*). In WT mice, total TAG, DAG, and FFA were not increased after only 6 hours following IR surgery, but several individual species were increased compared with sham operated mice (Figure 7*B–D*) (all lipids *P* < .05 WT sham vs WT IR 6 hours). Of note, all these species were significantly lower in AS-ATGL KO mice compared with WT mice following liver IRI. Furthermore, none of the lipid species measured were significantly increased in AS-ATGL KO mice following IRI, relative to sham mice (Figure 7*B–D* and Supplemental Table 1).

### Fatty Acid Treatment of Hepatocytes Worsens In Vitro IRI

**Figure 8:**
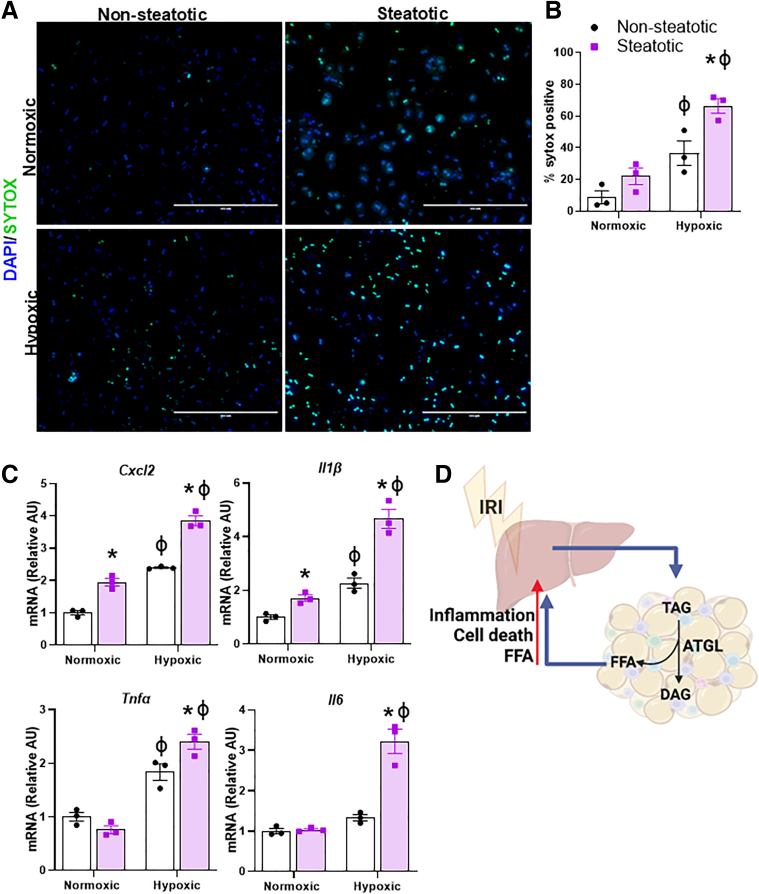
**Fatty acid treatment of hepatocytes worsens in vitro IRI.** (*A*) Representative images of DAPI- and Sytox-stained hepatocytes treated with vehicle (non-steatotic) or oleic acid and palmitic acid (steatotic) followed by exposure with IRI. (*B*) Quantitative scoring of Sytox-positive (dead) cells in non-steatotic or steatotic hepatocytes exposed to normoxic or hypoxic conditions. (*C*) Hepatocyte gene expression of *Cxcl2*, *Il1β*, *Tnfα*, and *Il6*. Values are mean ± SEM. n = 3 separate experiments, 3 wells per condition. ∗*P* < .05 between non-steatotic and steatotic under the same oxygen conditions. ϕ*P* < .05 between normoxic and hypoxic under the same lipid conditions. (*D*) Schematic representation of liver-adipose crosstalk in liver IRI.

To determine if FFAs were sufficient to worsen IRI, we isolated primary mouse hepatocytes from C57BL/6J mice and cultured them in the absence (non-steatotic) or presence of palmitic acid and oleic acid (steatotic). Hepatocytes were exposed to either normoxic or hypoxic conditions for 5 hours, followed by return to normoxic conditions for 5 hours. The addition of fatty acids significantly exacerbated in vitro ischemia reperfusion injury. Specifically, fatty acid treatment increased cell death and expression of genes encoding inflammatory cytokines and chemokines (Figure 8*A–C*). Thus, FFAs were sufficient to exacerbate liver IRI.

In summary, we have found that adipocyte-specific deletion of ATGL reduces circulating and hepatic content of FFAs. Suppressing adipose tissue release of FFAs by pharmacological inhibition or genetic deletion of ATGL attenuated liver IRI, whereas treatment of hepatocytes with fatty acids increased IRI. Thus, the beneficial effects of inhibiting adipocyte lipolysis in IRI are likely mediated by a reduction in potentially toxic circulating and hepatic FFAs (Figure 8*D*).

## Discussion

IRI is associated with significant morbidity and mortality. Although largely unavoidable in liver transplantation and other surgeries, there are no pharmacological interventions available to attenuate injury. Few studies have evaluated the role of interorgan crosstalk in liver IRI. In this study, we aimed to delineate the role of adipocyte lipolysis in liver IRI and determine whether inhibition of lipolysis would be a viable therapeutic target to attenuate IRI.

Adipose tissue stores excess energy in the form of TAG, and adipocyte lipolysis has a critical role in maintaining systemic homeostasis. Lipolysis is tightly regulated and is stimulated by various physiological conditions including starvation, exercise, or activation of the sympathetic nervous system resulting in norepinephrine release. Activation of β3 adrenergic receptors on adipocytes leads to ATGL mediated hydrolysis of TAG and the release of FFAs.^5^^,^^21^ Importantly, pharmacologic inhibition or genetic deletion of ATGL inhibits lipolysis and prevents a rise in blood FFAs in response to lipolytic stimuli.^12^^,^^13^ Adipose tissue dysfunction and excessive lipolysis are associated with pathological states that impact other organ systems such as the heart, skeletal muscle, and liver.^5^ Because the liver and other organs readily take up FFAs from the blood, excessive adipose tissue lipolysis can lead to ectopic deposition of fatty acids, generation of potentially bioactive lipid species, and can contribute to the development of insulin resistance and steatotic liver disease.^5^^,^^22^ However, the role of adipose-liver crosstalk in liver IRI has not been investigated.

In this study, we found that hepatic IRI is associated with enhanced adipocyte lipolysis, as evidenced by increased plasma FFAs and glycerol at 6 hours after IRI, which occurs before the accumulation of hepatic TAG observed at 24 hours.^19^ We found that inhibition of lipolysis using a chemical inhibitor of ATGL or genetic deletion of ATGL in adipocytes significantly reduced liver IRI, whereas hepatocyte-specific deletion of ATGL had no effect. Furthermore, improvement in liver IRI was accompanied by a significant reduction in circulating FFAs, suggesting that the improvement in liver IRI is likely due to decreased accumulation of potentially toxic FFAs in the liver. This is supported by cell culture experiments in which the addition of FFAs significantly worsens hepatocyte IRI in vitro. Inhibition of adipocyte lipolysis leads to a reduction in hepatic TAG and potentially lipotoxic intermediates such as DAG and FFAs. Taken together, these data suggest that inhibition of ATGL-mediated adipocyte lipolysis protects against liver IRI at least in part by reducing circulating FFAs and decreasing hepatic lipid content, thereby reducing liver inflammation and cell death. This is supported by previous studies, which have found that elevated circulating NEFAS and ectopic lipid accumulation can activate inflammatory pathways.^23^ Furthermore, others have reported that adipocyte lipolysis can modulate the hepatic transcriptome and lipidome,^15^^,^^16^ and we have shown that liver IRI induces global changes in the hepatic lipidome.^19^ Based on our findings and others, it is likely that these changes are mediated by enhanced adipocyte lipolysis. However, in the setting of severe acute injury such as IRI, the liver may not have the metabolic flexibility to adapt to rapid changes in lipid composition.

Our findings are in agreement with previous work suggesting that inhibition of ATGL in adipose tissue improves heart function in both models of acute myocardial injury and chronic heart failure.^18^^,^^24^^,^^25^ Although whole body ATGL-deficient mice have significant cardiomyopathy, mice with adipocyte-specific deletion of ATGL have preserved heart function and exhibit reduced cardiac injury and fibrosis compared with WT mice in multiple models of cardiac injury.^24^, ^25^, ^26^ Interestingly, Mathur et al^17^ found that mice deficient in adipocyte lipolysis were protected from ethanol-induced hepatic steatosis and oxidative stress, but not liver inflammation.^17^ In contrast, we did not find baseline differences in plasma ALT or hepatic infiltration of neutrophils. These differences are likely due to differences in mouse and liver injury models.

We postulate that the mechanism by which inhibition of adipose lipolysis mediates its beneficial effects in liver IRI is via reduction of lipotoxic metabolites. We have shown that exogenous FFAs worsened IRI in hepatocytes in vitro, but we cannot rule out that other complex lipids released from adipose tissue, such as DAGs and ceramides, can impact liver IRI in the in vivo setting. Furthermore, the adipocyte secretome includes lipids, adipokines, and exosomes, which can act as substrates, signaling molecules, or precursors for other metabolites.^27^ Future investigations will need to delineate the origin and role of specific adipose-derived metabolites.

A limitation of the current study is that the specific mechanism by which hepatic IRI leads to adipocyte lipolysis is unclear. It is probable that acute hepatic IRI leads to a sympathetic surge that contributes to adipocyte lipolysis because we observed increased HSL phosphorylation after IRI. However, we cannot rule out the possibility of humoral mediators released by injured hepatocytes that could potentially trigger adipocyte lipolysis in the setting of IRI. Tracer studies and expanded lipidomic and transcriptomic studies in plasma, adipose, and liver may provide more clarity. Although the protective effects of inhibiting lipolysis are likely mediated by reducing accumulation of lipotoxic metabolites, we have not yet identified a specific toxic lipid mediator. Additionally, our study only assessed one time point, but we and others have found hepatic lipid remodeling after IRI to be a dynamic process.^15^^,^^19^ Thus, it will be important to assess additional reperfusion time points in future studies.

Additionally, it would be important to translate these findings to humans undergoing liver transplantation. Given the hemodynamic changes intrinsically involved in liver transplantation and the potential need for vasopressor therapy, beta blockade, and prolonged inhibition, adipose tissue lipolysis is unlikely to represent a viable treatment or preventative strategy to mitigate IRI. However, brief inhibition using a short-acting inhibitor of lipolysis or prevention of conditions that may further exacerbate adipose lipolysis (ie, prolonged fasting) could represent viable therapeutic strategies in the setting of liver transplantation. Notably, a small molecule is available to specifically target human ATGL.^28^ As prolonged inhibition of ATGL may have unintended consequences, the ability to use a pharmacological inhibitor for a brief period may be beneficial in the setting of acute liver injury. Taken together, inhibition of adipose tissue lipolysis is a promising novel therapeutic strategy for the treatment and prevention of liver IRI, which can ultimately improve patient and graft outcomes.

## Materials and Methods

### Animals

C57BL/6J mice and *Pnpla2* floxed mice were purchased from Jackson Laboratory. *Pnpla2* floxed mice were crossed with C57BL/6J mice expressing *Cre* recombinase under the control of the Adiponectin or Albumin promoter to generate adipocyte specific *Pnpla2* knockout (KO) mice (AS-ATGL KO) and hepatocyte-specific *Pnpla2* KO mice (LS-ATGL KO), respectively. Littermate mice not expressing Cre were used as control (WT) mice for studies involving tissue-specific *Pnpla2* KO mice. Mice were housed in a specific pathogen-free animal facility at Washington University School of Medicine under a 12-hour light/dark cycle. All animal studies were approved by the Institutional Animal Use and Care Committee of Washington University and comply with the *Guide for the Care and Use of Laboratory Animals* as outlined by the National Academy of Sciences.

### Hepatocyte Isolation and In Vitro IRI

Primary mouse hepatocytes were isolated as previously described.^29^ Briefly, mice were deeply anesthetized with isoflurane and euthanized by cervical dislocation. Hepatocytes were isolated by perfusing livers with Hanks’ Balanced Salt Solution (HBSS) with EDTA followed by Dulbecco’s Modified Eagle Medium (DMEM) containing collagenase (Sigma Aldrich). Hepatocytes were plated overnight in collagen-coated 6-well plates with DMEM containing 10% fetal bovine serum (FBS) and antibiotics. The following day, a portion of cells were exposed to hypoxia by replacing media with a glucose-free, serum-free media (Gibco A1443001, ThermoFisher Scientific) and placed in a hypoxia incubator with 1% oxygen, 5% CO_2_, and 94% nitrogen. Cells exposed to normoxic conditions were treated with glucose- and serum-containing media and placed in a normoxic incubator. Cells were maintained in their respective incubator for 5 hours. At reperfusion, cells were removed from the incubator, and normoxic and hypoxic media were replaced with DMEM containing 10% FBS and antibiotics. After 5 hours of simulated reperfusion, cells were then harvested with Trizol for RNA isolation or stained with 4′,6-diamidino-2-phenylindole (DAPI) and SYTOX (ThermoFisher Scientific) for determination of cell death. Image J was used to quantify the percentage of SYTOX-positive cells per low powered field.

In some studies, cells were treated with vehicle (dimethyl sulfoxide [DMSO]) or Atglistatin (40 μM) after isolation and throughout the ischemia-reperfusion protocol. In other studies, hepatocytes were incubated with a mixture of palmitic acid (0.66 mM) and oleic acid (0.33 mM) (Sigma Aldrich) after isolation to induce lipid accumulation.

### Hepatic Ischemia Reperfusion Surgery

Hepatic ischemia was induced using a 70% ischemia model as previous described.^30^^,^^31^ Briefly, mice were anesthetized using isoflurane inhalation followed by laparotomy. Ischemia is established by cross-clamping the hepatic artery, portal vein, and bile duct distal to the branch point to the right lateral lobe. After 1 hour of ischemia, the atraumatic clamp was released to re-establish blood flow to the median and left lobes. Following 6 hours of recovery, animals were euthanized, and plasma and tissue samples were collected for analysis. Mice undergoing sham surgery underwent midline laparotomy without cross-clamping and remained under anesthesia for 1 hour.

In some studies, vehicle (olive oil) or Atglistatin 100 μmol/kg (Cayman Chemical Item No. 15284) was administered by oral gavage 1 hour prior to hepatic surgery. Timing and dose of Atglistatin was based on previous studies.^20^

### Plasma Parameters

Plasma ALT and AST were measured using a commercially available colorimetric kinetic assay (Teco Diagnostics) according to manufacturer’s instructions. Plasma NEFAs (Wako Diagnostics) and plasma glycerol (Sigma) were measured using commercially available kits according to manufacturer’s instructions.

### RNA Isolation and Gene Expression Analysis

Liver RNA was extracted with TRIzol and PureLink RNA Kit according to manufacturer’s instructions (Invitrogen, Thermo Fisher Scientific). Complementary DNA was synthesized using a high-capacity reverse transcription kit (Applied Biosystems). Quantitative real-time polymerase chain reaction was performed with Power SYBR Green (Thermo Fischer Scientific) with an ABI-Prism 7500 detection system (Applied Biosystems). Arbitrary units (AUs) of messenger RNA of the genes of interest were corrected to 36B4 expression. Primers were obtained from Integrated DNA Technologies. Primer sequences used in this study were: *36b4* (F: GCA GAC AAC GTG GGC TCC AAG CAG AT R: GGT CCT CCT TGG TGA ACA CGA AGC CC), *Abhd5* (F: AGA TGT GCC CTC AGG TTG GAC A, R: ATC TGG TCG CTC AGG AAA ACC C), *Cxcl2* (F: GCC AGA GGG GTT TCT GTC G R: GTT CGT GCC GCT AAA AGT CA), *Ccl2* (F: TTA AAA ACC TGG ATC GGA ACC AA R: GCA TTA GCT TCA GAT TTA CGG GT), *G0s2* (F: GCT AGT GAA GCT ATA CGT GCT GG R: GGA CTG CTG TTC ACA CGC TTC C), *Hig2* (F: AAG CAT GTG TTG AAC CTC TAC C R: TGT GTT GGC TAG TTG GCT TCT), *Il1b* (F: GCA ACT GTT CCT GAA CTC AAC T R: ATC TTT TGG GGT CCG TCA ACT), *Il6* (F: TAG TCC TTC CTA CCC CAA R: TTG GTC CTT AGC CAC TCC TTC), *Pnpla2* (F: ACA GTG TCC CCA TTC TCA GG R: CAC ATC TCT CGG AGG ACC AT), *Tnf* (F: CCC TCA CAC TCA GAT CAT CTT CT R: GCT ACG ACG TGG GCT ACA G).

### Western Blot Analysis

Liver protein extracts were obtained from the liver using RIPA lysis buffer (Cell Signaling Technology). Lysates were normalized to protein concentration, denatured, and run on NuPAGE precast gels (Thermo Fisher Scientific) and then transferred onto polyvinylidene fluoride membranes. Membranes were incubated with primary antibodies (1:1000 in 5% bovine serum albumin [BSA]) overnight and then probed with an anti-rabbit IRDye or anti-mouse IRDye secondary antibody (1:10,000, LI-COR). The blots were imaged using an infrared Odyssey developer (LI-COR). Primary antibodies were all obtained from Cell Signaling Technology: ATGL (#2138), HSL (#4107), phosphoHSL (#4139), and vinculin (#13901).

### Histology

A portion of the left lateral lobe was harvested at the time of sacrifice and placed in 10% neutral buffered formalin followed by 70% ethanol. The tissues were then embedded in paraffin, sectioned, and stained with hematoxylin and eosin (H&E) or MPO stain. Liver injury was evaluated by a pathologist blinded to the experimental conditions using Suzuki’s score, which combines assessment of necrosis, congestion, and vacuolization.^32^

### Liver TAG and NEFA Assay

Liver samples were homogenized in normal saline followed by addition of 1% sodium deoxycholate to solubilize lipids. Hepatic triglyceride and NEFA content were measured using commercially available colorimetric kits (Thermo Fisher Scientific and Wako Diagnostics).

### Mass Spectrometry and Lipid Quantification

Mass spectrometry studies were performed in the Metabolomics Facility at Washington University School of Medicine. Briefly, DAG and TAG were extracted with Bligh-Dyer method from 50 μL of homogenate. The DAG (21:0-21:0) and TAG (17:1-17:1-17:1) were used as internal standards for DAG and TAG, respectively. FFA was derivatized with 4-aminomethylphenylpyridium to improve mass spectrometric sensitivity. Quality control (QC) samples were prepared by pooling the aliquots of the study samples and were used to monitor the instrument stability. The QC was injected between every 7 study samples. Measurement of DAG and TAG was performed with a Shimadzu 20AD ultra fast liquid chromatography (UFLC) system coupled to an API4000 mass spectrometer operated in positive multiple reaction monitoring (MRM) mode. FFAs were detected with 4000QTRAP mass spectrometer coupled with Shimadzu 20AD UFLC system in positive MRM mode. Data processing was conducted with Analyst 1.6.3. The relative quantification data of lipids were reported as the peak area ratios of the analytes to the corresponding internal standards, and only lipid species with CV% < 15% in QC injections were reported.

### Adipose Tissue Lipolysis

Adipose tissue was excised and cultured as previously described.^33^ Briefly, eWAT and iWAT were excised from AS-ATGL KO mice and WT littermates, minced under sterile conditions, and maintained in culture for 48 hours prior to lipolysis assay. Tissue pieces were then washed and placed in wells containing basal media or media supplemented with isoproterenol to stimulate lipolysis. Aliquots of conditioned media were collected at 2 hours, and lipolysis was evaluated by measuring free glycerol and NEFA using a commercially available kit (Sigma and Wako Diagnostics) according to manufacturer’s instructions.

### Statistical Analysis

Statistical comparisons were made using a *t*-test or analysis of variance (ANOVA) with post-hoc Tukey, Sidak, or Bonferonni where appropriate. Statistical significance was defined as *P* < .05. Data are presented as mean ± standard error of the mean (SEM). Statistical analysis was performed using GraphPad Prism software.
